# Tuning the Magnetic Moment of Small Late 3d-Transition-Metal Oxide Clusters by Selectively Mixing the Transition-Metal Constituents

**DOI:** 10.3390/nano10091814

**Published:** 2020-09-11

**Authors:** Rodrigo H. Aguilera-del-Toro, María B. Torres, Faustino Aguilera-Granja, Andrés Vega

**Affiliations:** 1 Departamento de Física Teórica, Atómica y Óptica, Universidad de Valladolid, 47011 Valladolid, Spain; rodrigohumberto.aguilera@uva.es (R.H.A.-d.-T.); avega@fta.uva.es (A.V.); 2 Departamento de Matemáticas y Computación, Universidad de Burgos, 09006 Burgos, Spain; 3 Instituto de Física, Universidad Autónoma de San Luis Potosí, San Luis Potosí 78290, Mexico; faustino@ifisica.uaslp.mx

**Keywords:** magnetism, transition-metal oxide clusters, DFT calculations, structure, electronic properties, 75.75+a, 36.40Cg, 75.30.Pd, 75.50.-y

## Abstract

Transition-metal oxide nanoparticles are relevant for many applications in different areas where their superparamagnetic behavior and low blocking temperature are required. However, they have low magnetic moments, which does not favor their being turned into active actuators. Here, we report a systematical study, within the framework of the density functional theory, of the possibility of promoting a high-spin state in small late-transition-metal oxide nanoparticles through alloying. We investigated all possible nanoalloys A${}_{n-x}$B${}_{x}$O${}_{m}$ (A, B = Fe, Co, Ni; *n* = 2, 3, 4; 0≤x≤n) with different oxidation rates, *m*, up to saturation. We found that the higher the concentration of Fe, the higher the absolute stability of the oxidized nanoalloy, while the higher the Ni content, the less prone to oxidation. We demonstrate that combining the stronger tendency of Co and Ni toward parallel couplings with the larger spin polarization of Fe is particularly beneficial for certain nanoalloys in order to achieve a high total magnetic moment, and its robustness against oxidation. In particular, at high oxidation rates we found that certain FeCo oxidized nanoalloys outperform both their pure counterparts, and that alloying even promotes the reentrance of magnetism in certain cases at a critical oxygen rate, close to saturation, at which the pure oxidized counterparts exhibit quenched magnetic moments.

## 1. Introduction

For a long time, transition-metal oxide (TMO) nanoparticles (NP) have been the matter of intense research due to their relevance in a large variety of technological applications, such as those in medicine [1,2,3,4,5,6,7,8,9], new generation batteries [10,11,12], bactericidal agents [13,14,15], and catalytic processes [16,17,18,19,20,21]. TMO-NP are easy to obtain and cheap, and are being considered now as good candidates for the replacement of critical materials that are either harmful or scarce and expensive. Among their electronic properties, magnetism is one of the most relevant one. TMO-NP exhibit superparamagnetic behavior and low blocking temperature, even for sizes of tens of nanometers, due to their low magnetic anisotropy energy. Those characteristics make them useful, for instance, in magnetic resonance imaging and as magnetic markers, for which it is fundamental to avoid agglomeration within the environment where they have to act [22].

There is one thing in common for the efficiency of any magnetic NP, independently of the application in which it will be used: it should be as strongly magnetic as possible for it to be turned into active actuators. Unfortunately, this is precisely the weak point of TMO-NP in general, since TM–O interactions induce antiparallel (AP) magnetic couplings which render them to be in a low-spin state, and consequently to have a small total magnetic moment. To overcome this issue, attempts have been made to increase the total moment of TMO-NP by their doping with another element [23,24]. In this context, Szczerba et al. have experimentally investigated the doping of iron oxide nanoparticles with Zn with the goal of avoiding the spin missalignment, thereby enhancing the total moment [24]. The idea behind it is that Zn substitutes Fe atoms at tetrahedral sites, which are were the Fe atoms antiparallelly couple with the rest in the original iron oxide NP. Although this doping works by eliminating the harmful Fe atoms, the substituted Zn atoms do not contribute themselves to the total moment of the resulting doped NP, since they are nonmagnetic. However, if one could transform the AP couplings into P ones by means of a substitutional magnetic dopant instead of a nonmagnetic one, the total moment could be even larger. This is the main idea behind the present work.

TMO-NP of late transition-metal elements (Fe, Co and Ni) have been extensively investigated both experimentally and theoretically [25,26,27,28,29,30,31,32,33,34,35]. Photoelectron spectroscopy (PES) [36,37] allows one to determine the most abundant stoichiometries and cluster products resulting after fragmentation of a parent cluster. Ion mobility mass spectrometry (IMMS) [29,38] allows one to indirectly determine structural transition patterns as a function of cluster size. In the context of those type of experiments, DFT calculations have provided compelling evidence of which are the putative ground states (GS) of TMO-NP of different sizes. Our group has contributed to such knowledge by proposing GS of Co- and Ni-oxide cationic clusters [39,40] and Fe-oxide cationic clusters [41] after checking that those GS reproduced the most favorable fragmentation channels, the most abundant individuals in the mass spectra and the structural patterns consistent with IMMS. We have also investigated the neutral and anionic counterparts of those TMO-NP [39,40]. Once the GS configurations were benchmarked against the experimental results, we analyzed in detail which configurations are optimal from the magnetic point of view for their potential use as nanomagnets. An interesting and surprising result was that, although AP couplings characterize in general the TMO, leading to a low-spin states, we identified several TMO-NP in high-spin states with large total magnetic moments. There are even some TMO clusters with higher total moment than their non-oxidized transition-metal counterparts as a consequence of the robustness of the P magnetic couplings together with a non negligible contribution of the spin-polarization of the oxygen atoms to the total moment. We also verified that while increasing the d-band filling in the transition metal (TM) series (that is, while going from Fe to Ni), the paralell magnetic couplings become more robust against oxidation, although, in general, the AP couplings prevail nevertheless, particularly at high oxidation rates.

The above results mean that, in principle, there exists the possibility of enhancing the total magnetic moment of a TMO-NP by doping it substitutionally with a different TM element, preferably one on its right in the transition-metal series. The mechanism behind such improvement of the magnetic properties of the TMO-NP would be the promotion of P magnetic couplings, with a clear advantage over doping with nonmagnetic elements such as Zn: the total moment would be contributed to a large extent by the dopant. Moreover, there perhaps exists a particular TM mixing that leads to high-spin states for most oxidation rates which would imply the robustness of the total magnetic moment against the unavoidable environmental conditions. Exploring those aspects is appealing and this was the main objective of the present work, since no studies in this respect had been carried out to our knowledge, an exception being our study of oxidized FeCo dimers [42]. The results obtained in that study provide an illustrative example of what one could expect: the iron dimer oxide Fe${}_{2}$O${}_{m}$ is known to be in a low-spin state due to AP magnetic couplings [42]. In FeCoO${}_{m}$ clusters, the total moment oscillates as a function of *m* between high-spin states (characterized by P magnetic couplings) and low-spin states (characterized by AP couplings). Oxide clusters in the high-spin state retain the same total moment as the unoxidized FeCo dimer which largely exceeds the value of the Fe${}_{2}$O${}_{m}$ counterpart. In the context of extended magnetic alloys, Fe–Co ones are certainly interesting for applications in soft magnets. At 30–40% of Co content the Fe–Co alloy is the material with the highest saturation magnetization, exceeding that of pure Fe bcc [43]. We note that cooperative effects in other binary transition-metal systems have been already demonstrated.

In view of the information described above, we found it appealing to explore, in a systematic way, the possibility of inducing ferromagnetic-like order to promote a high-spin state in TMO-NP (TM = Fe, Co, Ni) by their doping with a different TM element of the list. For that purpose, we extended our previous work on oxidized FeCo dimers to all possible pure and bimetallic dimers (the smallest size), trimers and tetramers, allowing us to have a variety of stoichiometries and geometrical arrangements in the range of small sizes. The goal was to identify optimal mixed TM oxides from the magnetic point of view, and to extract qualitative trends, if possible, that might help to choose the best dopant for larger magnetic TMO-NP. Taking into account the database of geometries of the putative ground states and low-energy isomers of Co- and Ni-oxide clusters obtained in our previous works, and those recently obtained for Fe-oxide clusters, we determined the putative ground states of the mixed TM oxide clusters. Before carrying out the present study, a tasting was done for Cr clusters doped with Fe, Co and Ni. Cr is the TM element with the highest spin magnetic moment and the best candidate for a highly magnetic NP if ferromagnetic-like coupling can be promoted. Unfortunately, the AP couplings were robust against doping with a late 3d TM element. Since this was the main conclusion of this initial test, we did not find it necessary to publish the results obtained, but the reader can obtain more details upon request from the authors.

In the next section we describe our theoretical approach and computational method. The results are presented in Section 3. The main conclusions of the work are summarized in the last section.

## 2. Materials and Methods

We performed fully self-consistent DFT calculations using the SIESTA code [44], which solves the spin-polarized Kohn–Sham equations within the pseudopotential approach. For the exchange and correlation potential we used the Perdew–Burke–Ernzerhof form of the generalized gradient approximation (GGA) [45]. Our DFT setup, and in particular the chosen functional to describe exchange and correlation effects (the PBE functional), is similar to that employed in our previous studies of the pure metal oxide clusters [39,40,41,42]. In the one of [42], we conducted benchmark calculations on the diatomic molecules FeCo, FeO, CoO and O${}_{2}$, obtaining good agreement with experimental results. In those of [39,40] (devoted to Co and Ni oxide clusters, respectively), we obtained a rather good agreement with experiments regarding the fragmentation channels, for which good descriptions of ground states and relative energies are required. We employed norm-conserving scalar relativistic pseudopotentials [46] in their fully nonlocal form [47], generated from the atomic valence configurations $3d^{7}4s^{1}$, $3d^{8}4s^{1}$ and $3d^{9}4s^{1}$ for Fe, Co and Ni, respectively (with core radii 2.00, 2.32 and 2.44 a.u. for *s*, *p* and *d* orbitals), and $2s^{2}2p^{4}$ for O (with core radii 1.14 a.u.). Non-linear partial core corrections [48], which are known to be important for TM pseudopotentials, were included for Fe, Co and Ni at the core radius of 0.7 Å. Valence states were described using double-zeta basis sets for all atomic species. The energy cutoff used to define the real-space grid for numerical calculations involving the electron density was 250 Ry. The Fermi distribution function that enters in the calculation of the density matrix was smoothed with an electronic temperature of 15 meV. We used an energy criterium of 10${}^{-4}$ eV for converging the electronic density. The individual clusters were placed in a cubic supercell of 20×20×20 Å${}^{3}$, a size large enough as to make the interaction between the cluster and its replicas in neighboring cells negligible, allowing us to consider only the Γ point (k=0) when integrating over the Brillouin zone. The putative global minimum structures resulted from an unconstrained conjugate-gradient structural relaxation using the DFT forces; the input structures were relaxed until interatomic forces were smaller than 0.001 eV/Å.

We note that, although the size of the investigated clusters was small, the problem of locating the global minimum structures was still a formidable task due to the many variables involved which required use to consider many input configurations. First, except for the dimers, one has to consider the different homotops of the heteroatomic clusters, resulting from the different inequivalent positions of the atoms of the two transition-metal elements within the structural skeleton. Second, oxygen can be attached in top, bridge and hollow positions and between TM atoms, and also through atomic or molecular adsorption (particularly in the range of high oxygen concentration). Third, different total spin states have to be checked to find the spin magnetic moment of the putative ground state. A given spin state can result from different magnetic couplings within the system, P or AP in which case the spin homotops have to be also considered. On top of that, all variables are interconnected since they depend on each other. The protocol used for the structural search was as follows. First, we selected several low energy isomers of Fe, Co and Ni oxide clusters that were investigated in our previous works [39,40,41]. Details of the structural search for these pure TM oxide clusters can be found in those papers, and it is important to state here that an indirect support for them is the fact that consistency with fragmentation and ion mobility experiments was achieved. For all those configurations we mixed the different TM atoms to build inputs for the homotops of each oxide nanoalloy. This creates, in some cases, inequivalent positions for oxygen that in the pure TM oxide were equivalent by symmetry and therefore were not checked. For instance, in Fe${}_{2}$O, the oxygen atom can be attached only in two positions, in the bridge or on top of Fe, but in FeCoO oxygen can be also attached on top of Co. After adding to the database these new configurations arising from the inequivalent O positions, the next step was to perform, for each one, different DFT calculations for different spin isomers. In order to save computing time, good practice is to ensure selfconsistency in various steps. This allows one to not push to the end of accuracy those cases in which a weak selfconsistency is enough to: (i) determine that a given input has transformed into another input of the database; (ii) a given configuration energetically destabilizes with respect to others already checked.

## 3. Results and Discussion

We will discuss first the structural properties and stability of the binary TM oxides A${}_{n-x}$B${}_{x}$O${}_{m}$ (A, B = Fe, Co, Ni; *n* = 2, 3, 4; 0≤x≤n) with different oxidation rates, *m*. These properties are connected with their electronic structures and magnetic properties that will be discussed in a second subsection. There are several factors that contribute in determining the structural properties and stability in these binary TM oxide clusters, in particular the differences in binding energy of Fe, Co and Ni and the relative strengths of the metal–metal and metal–oxygen bonds. The Pauling scale electronegativities [49] of O, Fe, Co and Ni are 3.4, 1.83, 1.88 and 1.91, respectively, so we expected a non negligible electronic charge transfer from the metal atoms towards O atoms in these systems. This effect will strengthen metal–oxygen bonds due to the partial ionic contribution while weakening the metal–metal bonds. Concerning the magnetic properties, it is known that clusters of Fe, Co and Ni have P magnetic moments and larger per atom spin-polarization than their respective bulk counterparts (due to electron localizaton). However, due to the expected loss of electronic charge in the metal atoms, and the weakening of the metal–metal interaction, weakening of the tendency to P magnetic couplings was also expected. Moreover, the contribution of the spin-polarization of the oxygen atoms to the total moment may not be negligible. The interplay between all those factors depends on the composition and size, such that it is very difficult to anticipate which of the oxidized nanoalloys will exhibit the best figures of merit from the point of view of the magnetic properties without carrying out explicit calculations for each particular system.

### 3.1. Structural Properties and Stability

**Figure 1:**
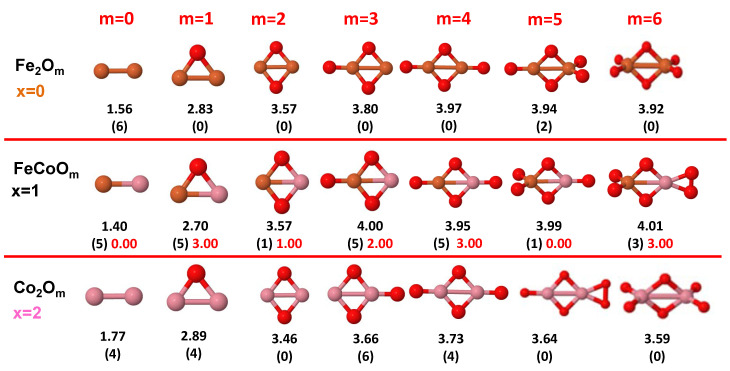
Putative global minimum structures of Fe${}_{2-x}$Co${}_{x}$O${}_{m}$, *x* = 0–2, *m* = 1–6. Numbers below structures are the binding energy per atom (in eV), numbers in parentheses are the magnetic moment (in $\mu_{B}$) and the third number (when present) is the excess magnetic moment (in $\mu_{B}$) defined in Equation (2). Fe atoms in brown color, Co atoms in pink and oxygen atoms in red.

**Figure 2:**
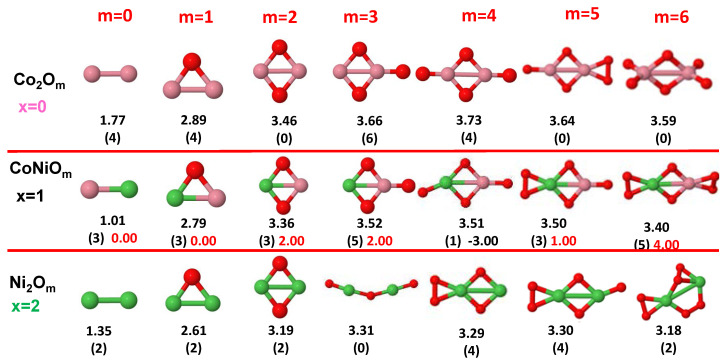
Putative global minimum structures of Co${}_{2-x}$Ni${}_{x}$O${}_{m}$, *x* = 0–2, *m* = 1–6. Numbers below structures are the binding energy per atom (in eV), numbers in parentheses are the magnetic moment (in $\mu_{B}$) and the third number (when present) is the excess magnetic moment (in $\mu_{B}$) defined in Equation (2). Co atoms in pink color, Ni atoms in green and oxygen atoms in red.

**Figure 3:**
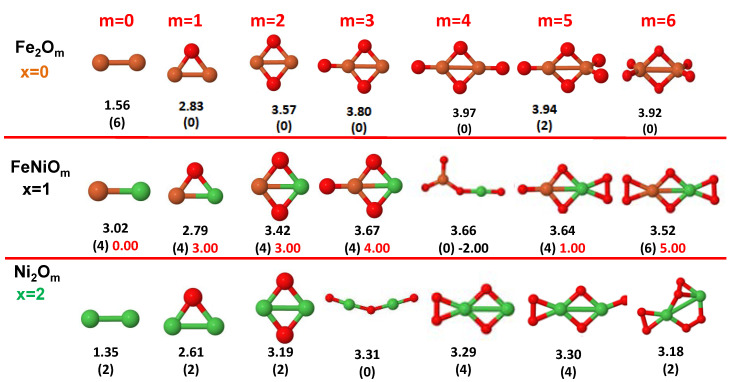
Putative global minimum structures of Fe${}_{2-x}$Ni${}_{x}$O${}_{m}$, *x* = 0–2, *m* = 1–6. Numbers below structures are the binding energy per atom (in eV), numbers in parentheses are the magnetic moment (in $\mu_{B}$) and the third number (when present) is the excess magnetic moment (in $\mu_{B}$) defined in Equation (2). Fe atoms in brown color, Ni atoms in green and oxygen atoms in red.

**Figure 4:**
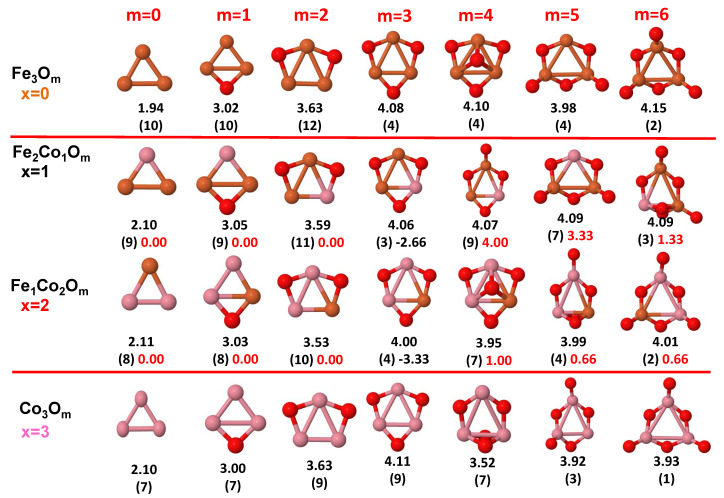
Putative global minimum structures of Fe${}_{3-x}$Co${}_{x}$O${}_{m}$, *x* = 0–2, *m* = 1–6. Numbers below structures are the binding energy per atom (in eV), numbers in parentheses are the magnetic moment (in $\mu_{B}$) and the third number (when present) is the excess magnetic moment (in $\mu_{B}$) defined in Equation (2). Fe atoms in brown color, Co atoms in pink and oxygen atoms in red.

**Figure 5:**
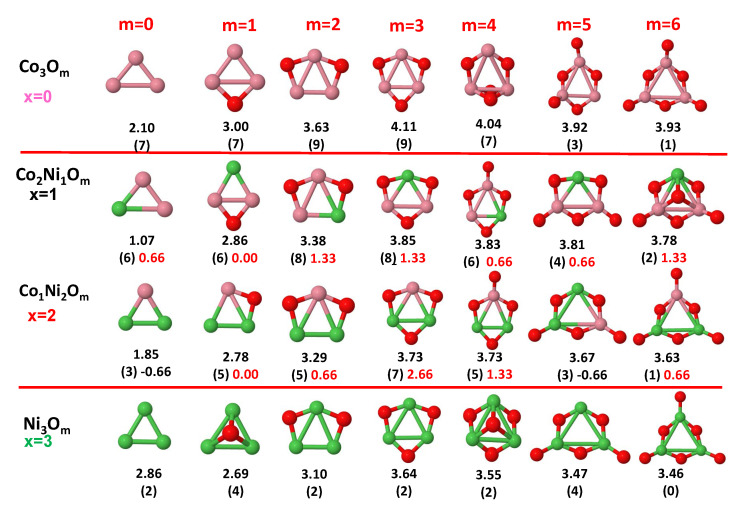
Putative global minimum structures of Co${}_{3-x}$Ni${}_{x}$O${}_{m}$, *x* = 0–2, *m* = 1–6. Numbers below structures are the binding energy per atom (in eV), numbers in parentheses are the magnetic moment (in $\mu_{B}$) and the third number (when present) is the excess magnetic moment (in $\mu_{B}$) defined in Equation (2). Co atoms in pink color, Ni atoms in green and oxygen atoms in red.

**Figure 6:**
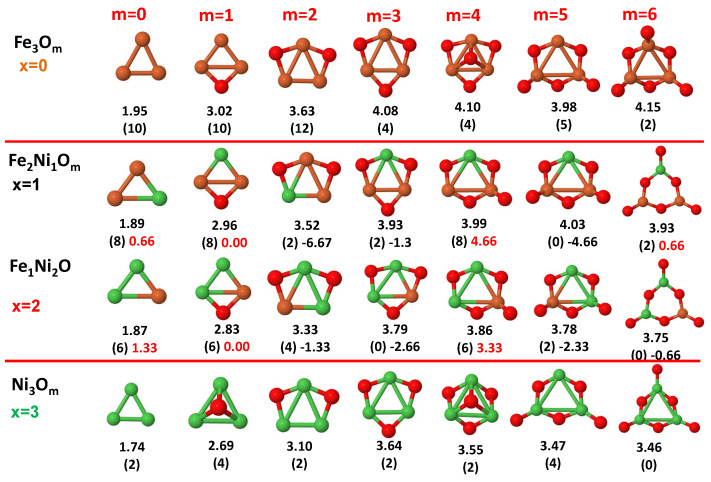
Putative global minimum structures of Fe${}_{3-x}$Ni${}_{x}$O${}_{m}$, *x* = 0–2, *m* = 1–6. Numbers below structures are the binding energy per atom (in eV), numbers in parentheses are the magnetic moment (in $\mu_{B}$) and the third number (when present) is the excess magnetic moment (in $\mu_{B}$) defined in Equation (2). Fe atoms in brown color, Ni atoms in green and oxygen atoms in red.

**Figure 7:**
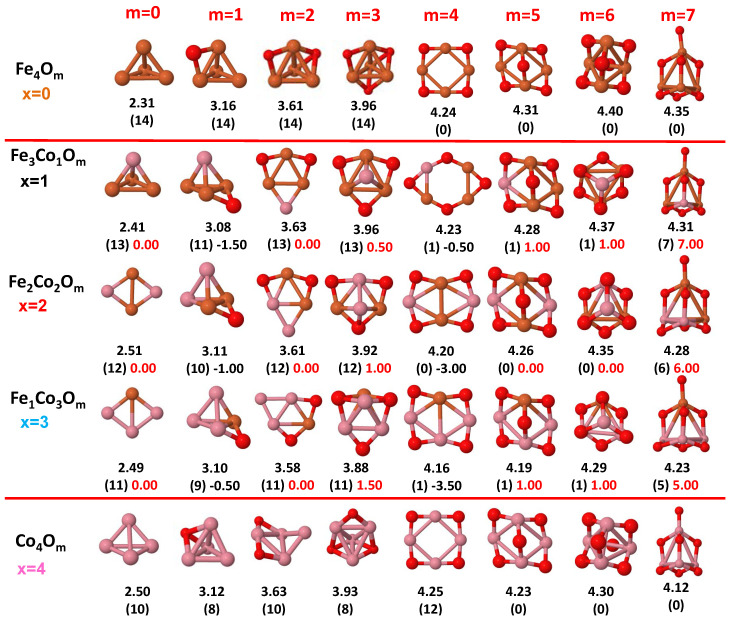
Putative global minimum structures of Fe${}_{4-x}$Co${}_{x}$O${}_{m}$, *x* = 0–2, *m* = 1–6. Numbers below structures are the binding energy per atom (in eV), numbers in parentheses are the magnetic moment (in $\mu_{B}$) and the third number (when present) is the excess magnetic moment (in $\mu_{B}$) defined in Equation (2). Fe atoms in brown color, Co atoms in pink and oxygen atoms in red.

**Figure 8:**
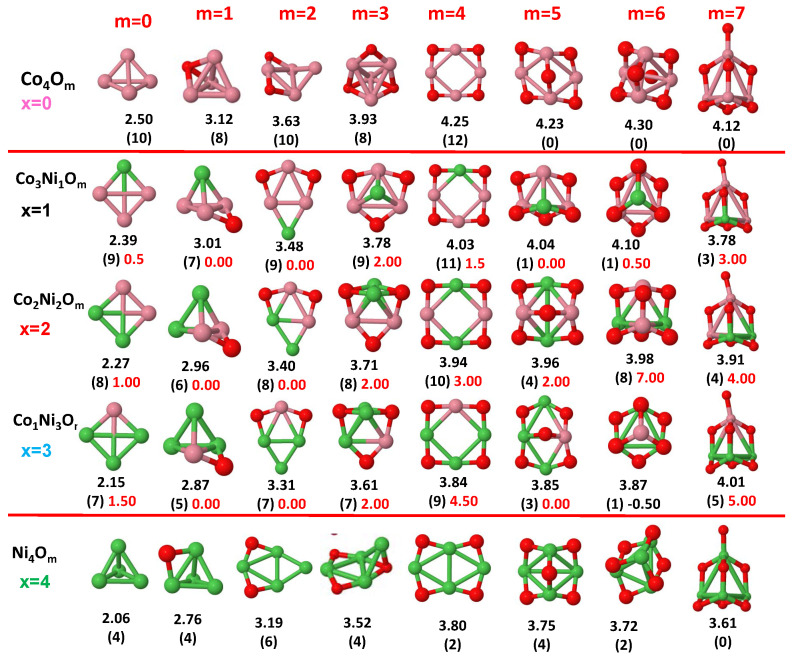
Putative global minimum structures of Co${}_{4-x}$Ni${}_{x}$O${}_{m}$, *x* = 0–2, *m* = 1–6. Numbers below structures are the binding energy per atom (in eV), numbers in parentheses are the magnetic moment (in $\mu_{B}$) and the third number (when present) is the excess magnetic moment (in $\mu_{B}$) defined in Equation (2). Co atoms in pink color, Ni atoms in green and oxygen atoms in red.

**Figure 9:**
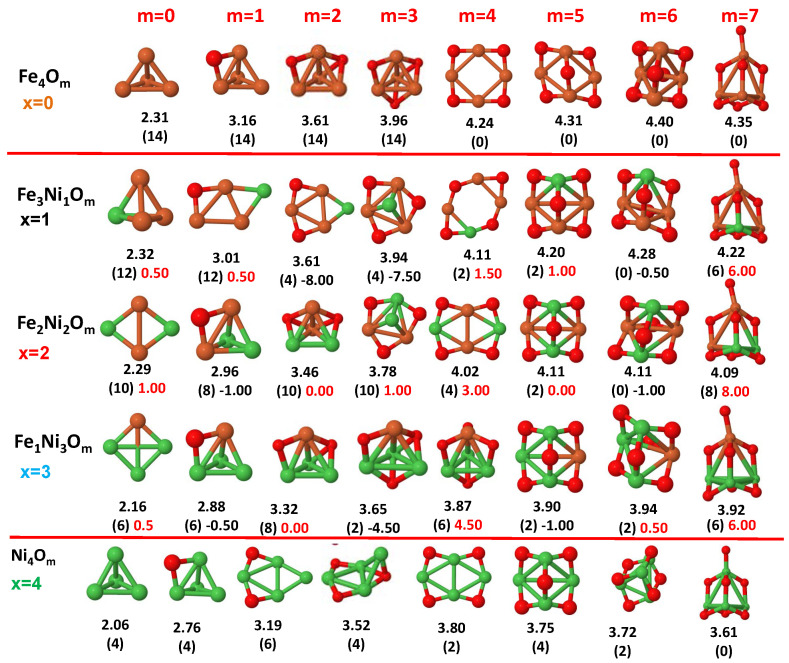
Putative global minimum structures of Fe${}_{4-x}$Ni${}_{x}$O${}_{m}$, *x* = 0–2, *m* = 1–6. Numbers below structures are the binding energy per atom (in eV), numbers in parentheses are the magnetic moment (in $\mu_{B}$) and the third number (when present) is the excess magnetic moment (in $\mu_{B}$) defined in Equation (2). Fe atoms in brown color, Ni atoms in green and oxygen atoms in red.

**Figure 10:**
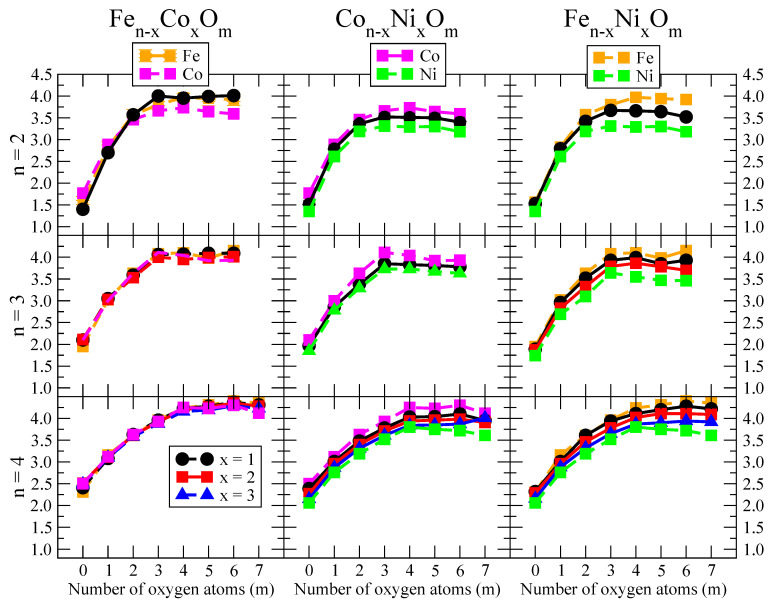
Binding energy per tom A${}_{n-x}$B${}_{x}$O${}_{m}$ clusters (A, B = Fe, Co, Ni; 0 ≤ *x* ≤ *n*; *m* = 1–7).

The putative global minimum structures of A${}_{n-x}$B${}_{x}$O${}_{m}$ (A, B = Fe, Co, Ni; 0≤x≤n), with *m* up to oxygen saturation, are shown in Figure 1, Figure 2 and Figure 3, Figure 4, Figure 5 and Figure 6 and Figure 7, Figure 8 and Figure 9 for *n* = 2, 3 and 4, respectively. In Figure 10 we plot the average binding energy per atom of the corresponding binary TM oxide clusters as a function of the oxidation rate. This quantity is calculated as:

(1) $$E_{b}\left(A_{n-x}B_{x}O_{m}\right)=\frac{-E\left(A_{n-x}B_{x}O_{m}\right)+\left(n-x\right)E\left(A\right)+xE\left(B\right)+mE\left(O\right)}{n+m}$$

where E(S) is the total energy of system *S*, and the values are also given below the structures shown in Figure 1, Figure 2, Figure 3, Figure 4, Figure 5, Figure 6, Figure 7, Figure 8 and Figure 9.

Some general trends can be inferred regarding the geometrical structures. Most pure and oxidized TM dimers and trimers, and their related mixtures, have a lineal and triangular subcluster of TM atoms, respectively. The exceptions to this trend are Ni${}_{2}$O${}_{3}$ with a bent linear structure with exclusively metal–oxygen bonds (similar to that found by Aguilera del Toro et al. [50] in In${}_{2}$O${}_{3}$), and FeNiO${}_{4}$ which does not have a Fe–Ni bond either. For tetramers (n=4), a competition between three-dimensional and planar structures is found. All pure TM clusters have a tetrahedral structure, but their respective non-oxidized nanoalloys have a planar rhombic structure in some cases, such as Fe${}_{2}$Co${}_{2}$, Fe${}_{1}$Co${}_{3}$ and all Co–Ni nanoalloys for n=3, Fe${}_{2}$Ni${}_{2}$ and Fe${}_{1}$Ni${}_{3}$. Although in oxidized TM clusters [39,40,41], the tetrahedron prevails for m=1,6,7, TM${}_{4}$O${}_{5}$ (TM${}_{4}$O${}_{4}$) oxides show a slightly bent rhombus (planar ring-like structure). Moreover, for Ni${}_{4}$O${}_{3}$ and Co${}_{4}$O${}_{2}$, an open tetrahedron is obtained, becoming a rhombus for Ni${}_{4}$O${}_{2}$. For the rest of cases, the tetrahedral TM subcluster is preserved. We have found that, in general, more planar structures appear for oxidized nanoalloys than for their bare counterparts. Thus, for m=2, all oxidized nanoalloys are planar except Fe${}_{2}$Ni${}_{2}$O${}_{2}$ and Fe${}_{1}$Ni${}_{3}$O${}_{2}$, for which the tetrahedron found for Fe${}_{4}$O${}_{2}$ still remains. In some cases, changes are found with respect to the tetrahedral structure for oxidized nanoalloys: Fe${}_{1}$Co${}_{3}$O${}_{3}$, Co${}_{2}$Ni${}_{2}$O${}_{3}$ and Co${}_{1}$Ni${}_{3}$O present a 3D structure consisting of two triangles sharing the same side, halfway between a bent rhombus and a tetrahedron.

**Figure 11:**
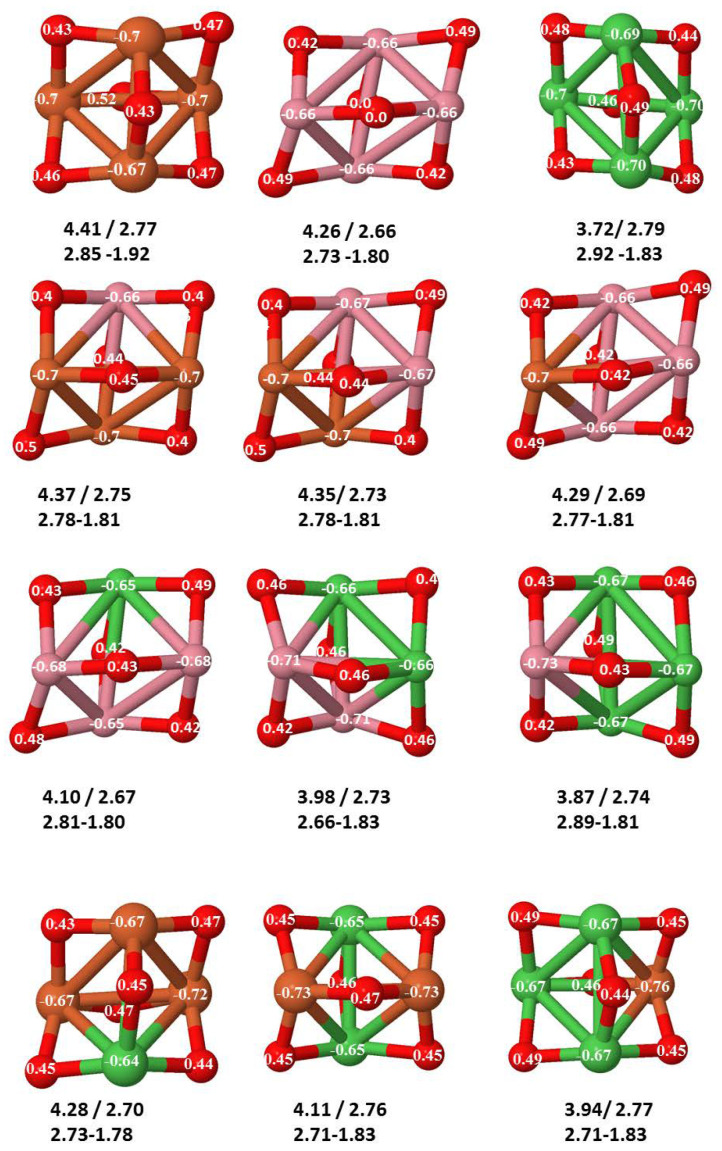
(Color online) Ground states of A${}_{4-x}$B${}_{x}$O${}_{6}$ (A, B = Fe, Co, Ni; *x* = 0–4) oxides. The excess (or defect) of the electronic charge for Fe, Co and O is given for each atom. Positive (negative) numbers indicate excess (defect). Below each of the structures, there are two rows of numbers. In the first one, binding energy per atom (in eV) and average electronic charge transfer (in *e*) are given. In the second one, both average TM–TM and TM–O distances are given. Fe atoms in brown color, Co atoms in pink, Ni atoms in green color and oxygen atoms in red.

The following general trends can be also inferred from the results shown in Figure 1, Figure 2, Figure 3, Figure 4, Figure 5, Figure 6, Figure 7, Figure 8, Figure 9 and Figure 10:

(i) The oxygen atoms tend to occupy bridge sites between the TM atoms (m≤n for n=2,3 and m≤6 for n=4), and when bridge sites are saturated, O atoms tend to occupy top sites (m=3,4 for n=2, m=4,5,6 for n=3 and m=7 for n=4).
(ii) The oxygen binds preferentially with Fe atoms. When the oxygen rate increases, the next preferential coordination is Co and finally Ni.
(iii) The net electronic charge in the O atoms, resulting from the charge transfer from the metal atoms, favors an uniform distribution of the O atoms in the cluster.
(iv) At oxidation rates where the number of O atoms equals the number of metal atoms, ring-like structures are formed for all sizes considered in this work. In this atomic configuration all metal–metal bonds are mediated by oxygen. This trend was already discussed in our previous works for the pure Fe, Co and Ni oxidized clusters [39,40,41], and it has been also discussed by other groups for other systems [33,51] in this small size range. We now demonstrate that these ring-like structures are preserved in the TM oxidized nanoalloys, except for Fe${}_{1}$Ni${}_{3}$O${}_{4}$, for which a tetrahedral structure is obtained, as stated above.
(v) We considered up to *m* = 6 or 7 oxygen atoms which is enough to analyze the transition from atomic to molecular adsorption in the case of the TM dimers (not yet in the trimers and tetramers). When the oxygen concentration is high enough, oxygen starts to be adsorbed molecularly, preserving short O–O inter-atomic distances. The critical oxygen concentration for this to occur lowers while going from Fe to Co and Ni, as it can be clearly seen in the structures of the oxidized dimers in Figure 1, Figure 2 and Figure 3: while in Fe${}_{2}$O${}_{m}$ no molecular adsorption takes place for m≤6, in Co${}_{2}$O${}_{m}$ occurs earlier (for m=5) and in Ni${}_{2}$O${}_{m}$ it starts even earlier (for m=4). Concerning mixed oxidized clusters, in FeCoO${}_{m}$ no molecular adsorption takes place for *m* = 6 while in CoNiO${}_{m}$ and FeNiO${}_{m}$ it already occurs for *m* = 5. The molecular adsorption is a manifestation of oxygen saturation of the system and its emergence at different oxygen concentrations for the different TM compositions is connected with the relative strength of the respective metal–oxygen bonds. The stronger the metal–oxygen binding is, the more oxygen is bound in an atomic form. In Table 1 we give, as a reference, the binding energies of Fe–O, Co–O and Ni–O, along with those of the different TM dimers. The metal–oxygen binding lowers for TM oxides while going from Fe to Co and Ni, as electronic charge transfer decreases and bond distance slightly increases.
(vi) The metal–metal binding also lowers while going from Fe to Ni (see Table 1). However, the Co dimer has the highest binding (and the lowest bond distance). General structural trends are also consistent with the binding energy per atom plotted in Figure 10 for the oxides. Consequently, for TM oxide dimers, and in connection with the discussion of the oxygen saturation, we note a shift to lower *m* in the maximum of binding energy while going from Fe to Co and Ni which means that adsorption of more oxygen atoms beyond this critical value of *m* does not increase the binding, because the metal–metal bonding weakens as *m* increases. The weakening of the metal–metal bonding is manifested in the less compact metal skeleton (lower average metal–metal inter-atomic distance) that results as *m* increases.
  The upper panel of Figure S1 shows the increase on the average metal–metal distance for the Co${}_{2}$Ni${}_{1}$O${}_{m}$ (0≤m≤6) nanoalloys. This distance increases until the triangular structure has all the oxygen atoms on the bridge positions (m=3), the increase being higher when both bridge and top positions are occupied by oxygen (m=6), where an open triangular subcluster is found. The average Co–Co and Ni–Co distances are also shown. Both increase also as the oxygen rate increases, with the Co atoms remaining somewhat further apart from each other than the Ni atoms, for a given oxidation rate. The lower panel of Figure S1 displays the average distance between the TM atoms and the O atoms located on bridge sites, which slightly decreases as the amount of oxygen decreases, mainly due to the reduction of the average Co–O distance. As the triangular subcluster opens up, the TM atoms are nearest to the bridge O atoms.
  Moreover, the results for the binding energies indicate that the higher the concentration of Fe in the TM oxide clusters, the higher their absolute stability, which may be important for practical purposes. On the other hand, the higher the Ni content, the lower the exothermic character of oxidation, or in other words, the less prone to oxidation the nanoparticle will be, which may also be important for practical purposes.
(vii) The relative differences of electronegativities between the TM atoms, although small, also support this trend. Electronegativity increases while going from Fe to Ni so that the tendency towards electron donation to O should be stronger in Fe atoms. Electronic charge transfers also corroborate this fact. Figure 11 illustrates an example of the correlation between the binding energies and the electronic charge transfer from the TM atoms to the O ones for all pure oxides and oxidized nanoalloys for a given oxidation rate, A${}_{4-x}$B${}_{x}$O${}_{6}$ (A, B = Fe, Co, Ni, 0≤x≤4 and m=6). Moreover, the binding energy per atom and the average electronic charge transfer are plotted in Figure S2 for Fe${}_{4-x}$Co${}_{x}$O${}_{6}$, Co${}_{4-x}$Ni${}_{x}$O${}_{6}$, and Fe${}_{4-x}$Ni${}_{x}$O${}_{6}$ as a function of *x*. The binding energy decreases for pure TM oxides from Fe${}_{4}$O${}_{6}$ to Co${}_{4}$O${}_{6}$ and from Co${}_{4}$O${}_{6}$ to Ni${}_{4}$O${}_{6}$, and it also decreases for any type of oxidized mixture, as the concentration (*x*) of the B constituent increases. For Fe–Co nanoalloys, the decrease of binding energy is the lowest among all mixtures, the highest being for Fe–Ni nanoalloys. Regarding the electronic charge transfer, Ni atoms are less prone to donate electrons to O due to their higher electronegativity. This is illustrated in the local electronic charges on the atoms of the respective structures shown in the Figure 11. In addition, as the concentration of Ni increases both in Co–Ni and Fe–Ni mixtures, each of theTM atoms (Fe, Co, Ni) increases its electronic donation, and consequently, the total transferred electronic charge becomes higher (see Figure S2); and this fact is reflected in a weakening of the bonding between the TMs, and therefore, in a decrease of the binding energy. However, in the case of FeCo mixtures, the binding energies do not vary significantly as the concentration of Co(*x*) increases, because the electronic transfer remains almost constant. Since the electronic transfer of Fe is higher than that of Co, and there is a lesser concentration of the former, the total electronic charge transfer from the TM atoms to oxygen decreases slightly instead of increasing as in the rest of the mixtures. Consequently, the binding energies in Fe–Co mixtures are more similar. That is, the oxides scarcely loose stability upon the substitution of Fe by Co. This fact, together with the magnetism that these mixtures present, makes them remarkable. In Figure 11, both average TM–TM and TM–O distances are also provided. The former are almost constant for both Fe–Co and Fe–Ni mixtures, and in the case of Co–Ni mixtures for x=2, the TM subcluster is somewhat more compact. The TM–O distances have little variation.

Up to which extent those facts have consequences on the magnetic properties of these systems is discussed in the next subsection.

Although several general structural and energetic trends have been found and rationalized in terms of relatively simple arguments, quantum confinement effects and the non scalability of physical and chemical properties inherent to the nanoscale lead to certain anomalies or unexpected structures that depart from the general behavior. This confirms again the need of performing explicit calculations for each individual system at the nanoscale in order to capture the details. Example of some peculiarities are, for instance, for dimers, Ni${}_{2}$O${}_{6}$ with three oxygen molecules, and the related to the geometrical changes above discussed. In the case of oxidized trimers: (i) Fe${}_{1}$Co${}_{2}$O${}_{m}$ (m=4,5) and Fe${}_{2}$Co${}_{1}$O${}_{6}$ oxides with two bound oxygen atoms on the same bridge, in the same way that they were found in Co${}_{3}$O${}_{4}$ oxide [39]; (ii) Co${}_{2}$Ni${}_{1}$O${}_{6}$ oxide, with the sixth oxygen atom on hollow site instead of top position after saturating all bridge sites, similar to the fourth one for Ni${}_{3}$O${}_{4}$ and Fe${}_{3}$O${}_{4}$ oxides. For tetramers, in addition to the geometric changes already described: (i) Fe${}_{3}$O${}_{4}$ and Ni${}_{3}$O${}_{4}$ with an oxygen atom in hollow site instead of an top after saturating all bridge sites; (ii) Co${}_{3}$O${}_{4}$ with two bound oxygen atoms on the same bridge.

### 3.2. Magnetic Properties

Small clusters of late 3d TM atoms are superparamagnetic with very low magnetic anisotropy energy [52,53,54]. However, the parallel magnetic couplings and relatively high spin polarization due to electron localization make them have high total magnetic moments which is their magnetic figure of merit. Nanoparticles with high total moments and low magnetic anisotropy are relevant, among others, for applications in which rotation of the nanoparticles should not affect the orientation of the moment when an external field is applied. This behavior is adequate for designing magnetic markers in nanomedicine, where particle aglomeration is also to be avoided. TM oxide clusters are among the most relevant magnetic nanoparticles in this context but, besides the absolute stability already discussed in the previous subsection, it is important to achieve (i) high total magnetic moments, and (ii) robustness of the total magnetic moment (mainly contributed by the TM atoms) against oxidation. The discussion of the present subsection is oriented towards the seeking of those binary TM oxide clusters with particularly high total moments among the investigated ones A${}_{n-x}$B${}_{x}$O${}_{m}$; the determination of those compositions that lead to more robust magnetic moments against oxidation; and finally, the seeking of general trends that could be valid for larger sizes.

**Figure 12:**
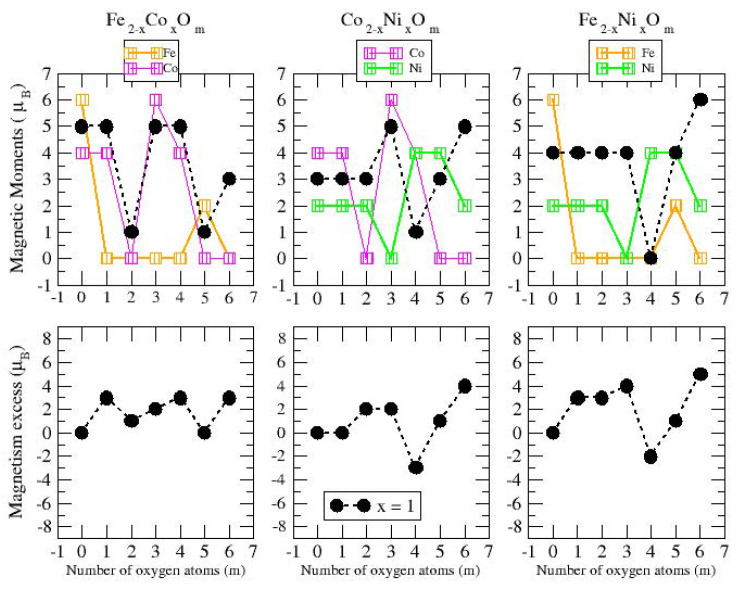
(**upper pannels**) Total magnetic moments of A${}_{2-x}$B${}_{x}$O${}_{m}$ clusters (A, B = Fe, Co, Ni; *x* ≤ 2; *m* = 1–6). Brown, pink and green curves correspond, respectively, to Fe${}_{n}$O${}_{m}$, Co${}_{n}$O${}_{m}$ and Ni${}_{n}$O${}_{m}$ oxides. Black curves correspond to nanoalloys with *x* = 1. (**lower panels**) Magnetism excess of ABO${}_{m}$ nanoalloys (A, B = Fe, Co, Ni; *m* = 1–6).

**Figure 13:**
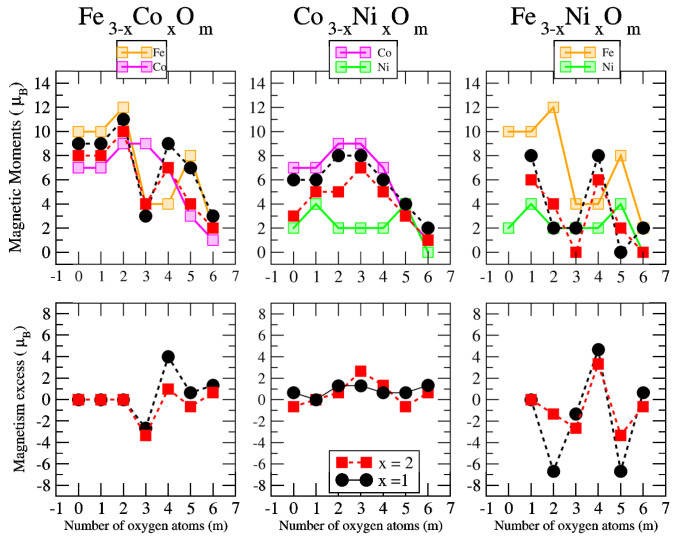
(**upper pannels**) Total magnetic moments of A${}_{3-x}$B${}_{x}$O${}_{m}$ clusters (A, B = Fe, Co, Ni; 0 ≤ *x* ≤ 3; *m* = 1–7). Brown, pink and green curves correspond, respectively, to Fe${}_{n}$O${}_{m}$, Co${}_{n}$O${}_{m}$ and Ni${}_{n}$O${}_{m}$ oxides. Black and red curves correspond to nanoalloys with *x* = 1, 2, respectively. (**lower panels**) Magnetism excess of A${}_{3-x}$B${}_{x}$O${}_{m}$ clusters (A, B = Fe, Co, Ni; 1 ≤ *x* ≤ 2; *m* = 1–7).

**Figure 14:**
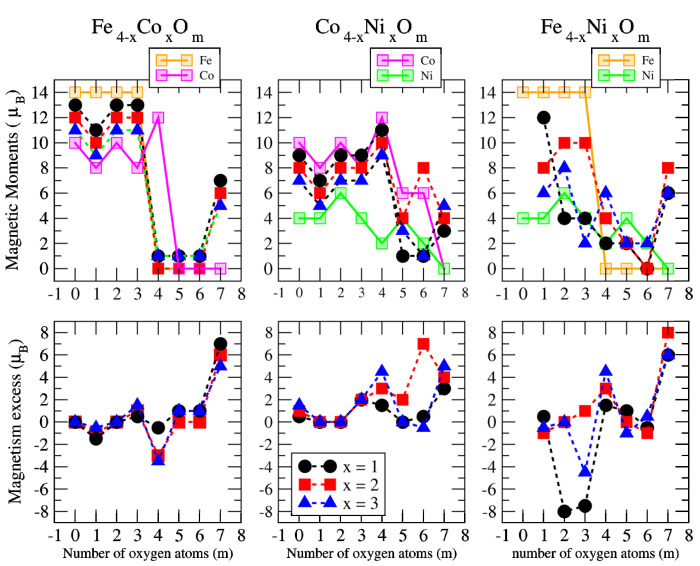
(**upper panels**) Total magnetic moments of A${}_{4-x}$B${}_{x}$O${}_{m}$ clusters (A, B = Fe, Co, Ni; 0 ≤ *x* ≤ 4; *m* = 1–7). Brown, pink and green curves correspond, respectively, to Fe${}_{n}$O${}_{m}$, Co${}_{n}$O${}_{m}$ and Ni${}_{n}$O${}_{m}$ oxides. Black, red and blue curves correspond to nanoalloys with *x* = 1, 2, 3, respectively. (**lower panels**) Magnetism excess of A${}_{4-x}$B${}_{x}$O${}_{m}$ clusters (A, B = Fe, Co, Ni; 1 ≤ *x* ≤ 3; *m* = 1–7).

The total magnetic moment of A${}_{n-x}$B${}_{x}$O${}_{m}$ (A, B = Fe, Co, Ni; 0≤x≤n) as a function of the oxygen concentration *m* is shown in the upper panels of Figure 12, Figure 13 and Figure 14 for *n* = 2, 3 and 4, respectively.

As a simple measure of the benefit of mixing TM atoms from the magnetic point of view, we define the excess magnetic moment or magnetic excess, $\mu_{exc}$, which in our systems is calculated as:

(2) $$\mu_{exc}\left(A_{n-x}B_{x}O_{m}\right)=\mu \left(A_{n-x}B_{x}O_{m}\right)-\frac{n-x}{n}\mu \left(A_{n}O_{m}\right)-\frac{x}{n}\mu \left(B_{n}O_{m}\right)$$

where μ(S) is the total magnetic moment of the system *S* in its ground state. Thus, $\mu (A_{n}O_{m})$ and $\mu (B_{n}O_{m})$ are the total moments of the oxide clusters of TM elements, *A* and *B*, in their respective global minimum structures at the oxygen concentration *m*. The excess magnetic moment is zero for TM oxide clusters of a single TM element by definition. In the rest of the cases, an excess magnetic moment equal to zero means that the mixture is an ideal mixture. The ideal mixture of $A_{n-x}B_{x}O_{m}$ would follow a simple Vegard law, according to which the total moment of the oxide nanoalloys follows a linear behavior connecting the magnetic moments of the pure oxide clusters, $A_{n}O_{m}$ and $B_{n}O_{m}$, obeying the quantization of the total spin. Positive excess magnetic moments indicate that the formation of the corresponding oxidized nanoalloy is magnetically favorable as compared to an ideal mixture, and often presents a magnetism even higher than that associated with the two pure systems. We note that similar magnitudes are often used in the analysis of energetic trends or the compactness of nanoalloys (excess energy [55] and excess radius [56], respectively), but to our knowledge, the excess magnetic moment was not defined. In the lower panels of Figure 12, Figure 13 and Figure 14, we plot the excess magnetic moment of the binary TM oxide nanoalloys, as a function of the oxygen concentration *m*.

#### 3.2.1. A-B Non-Oxidized Nanoalloys; A, B = Fe, Co, Ni

Before discussing the results for the oxidized mixtures, $A_{n-x}B_{x}O_{m}$, let us analyze the total magnetic moments and the values of the excess magnetic moment when *m* = 0, that is, for the bare $A_{n-x}B_{x}$ nanoalloys without the presence of oxygen. The preferred Fe–Fe magnetic coupling is parallel and Fe${}_{n}$ (*n* = 2–4) clusters show the highest magnetic moments equal to 6 $\mu_{B}$, 10 $\mu_{B}$ and 14 $\mu_{B}$, respectively. The Ni${}_{n}$ (*n* = 2–4) clusters, due to the lowest spin polarization, present the smallest values, equal to 2 $\mu_{B}$, 2 $\mu_{B}$ and 4 $\mu_{B}$, respectively, despite their ferromagnetic-like coupling, the Co${}_{n}$ (*n* = 2–4) clusters being those with the intermediate values, equal to 4 $\mu_{B}$, 7 $\mu_{B}$ and 10 $\mu_{B}$, respectively. In the case of non-oxidized Fe–Co alloys, the magnetic moments obtained follow the simple Vegard law with indexes equal to zero for all sizes studied here (*n* = 2–4). This behavior is also followed by the rest of the dimers (n=2) regardless the transition metal. The Fe–Ni nanoalloys with *n* = 3–4, although having lower moments than the corresponding Fe–Co ones, present higher excess magnetic moments (see Figure 11, Figure 12 and Figure 13, for m=0). The Co–Ni nanoalloys (particularly for n=4) exhibit the highest positive excess magnetic moments between 0.5 and 1.5. (Figure 13, for m=0).

#### 3.2.2. Fe–Co Oxided Nanoalloys

Among the late 3d elements, Fe has the most d-holes in its active valence states. This means that it has the largest local magnetic moments among the late TM elements but, at the same time, AP couplings are easier to be promoted in Fe systems than in Co and Ni ones. Electron transfer to O makes Fe electronically approach Mn, which already exhibits AP couplings in certain atomic arrangements and weakens the Fe–Fe interaction and the P magnetic couplings reminiscent of the Fe bulk ferromagnetism. This is the mechanism through which oxidation quenches the moment of the Fe dimer for nearly all oxidation rates *m* (see Figure 11), and strongly reduces those of the trimer (Figure 12) and tetramer (Figure 13) except for the lowest *m* in both cases, and also for m=5 in the case of dimers and trimers. This reentrance of μ at relatively high oxidation rates is an unexpected magnetic trend related with the particular structural arrangement and the strengthen of the Fe–O interaction to the point that the magnetic coupling between Fe and O in that particular geometry forces the O mediated Fe–Fe magnetic coupling to be P. This has been discussed in detail in our previous paper, where we show that further oxidation quenches the magnetic moment again [41]. Therefore, a general trend is that the Fe cluster must reach a critical size and metal–metal coordination to retain the P couplings under oxidation. When this happens (Fe${}_{3}$O${}_{m}$ with m<3, and Fe${}_{4}$O${}_{m}$ with m<4) we find the TM oxides with the maximum total moment among all oxidized nanoalloys investigated here—that is, those not further enhanced by any mixing with Co or Ni—and also larger than those of any Co–Ni oxide nanoalloy of the same size. However, another general trend is that the magnetic moment of iron oxide clusters is not robust against oxidation.

As compared to iron clusters, those of cobalt approach more the magnetic saturation due to the less d-holes in the active band states, which render them more ferromagnetic-like, although with less spin-polarization than their Fe counterparts. It is for this reason that Co oxide clusters retain, in general, a magnetic moment at oxidation rates at which Fe oxide clusters not, or they have a larger moment despite the less number of d-holes. Examples of this are Co${}_{2}$O${}_{m}$ with m=1,3,4; Co${}_{3}$O${}_{m}$ with m=3,4; Co${}_{4}$O${}_{m}$ with m=4. In particular, ring-like structures with *n* = 3–4 result in ferromagnets with magnetic moments equal to 9 $\mu_{B}$ and 12 $\mu_{B}$ [39], respectively, whereas their Fe counterparts exhibit an antiferromagnetic coupling [41] with magnetic moments of 4 $\mu_{B}$ and 0 $\mu_{B}$, respectively. The reentrance or punctual enhancement of the moment at certain high oxidation rates observed in Fe oxide clusters is not found, however, in the Co oxides, at least up to *m* = 7. It is for this reason that at those particular oxidation rates discussed in the previous paragraph, Fe oxide clusters exhibit a larger magnetic moment than their Co counterparts (Fe${}_{2}$O${}_{5}$, Fe${}_{3}$O${}_{5}$).

Combining the stronger tendency of Co towards P couplings with the larger spin polarization of Fe is particularly beneficial in the case of dimers (see left panel of Figure 11) where, for m=1,2,4 and 6, FeCoO${}_{m}$ has larger total moment than both Co${}_{2}$O${}_{m}$ and Fe${}_{2}$O${}_{m}$ (in these Fe oxides the moment is quenched except for m=5, where Fe–Co mixture show a linear magnetic behavior). This is reflected in the excess magnetic moment which is positive and high in dimers and with maxima in most of those values of *m*. The moment is also quenched in the cobalt oxide dimer at large oxidation rates (for m=5,6), but Fe–Co mixing leads to magnetic ground states even at those oxidation rates. For m=6, where the magnetic moment of Fe${}_{2}$O${}_{6}$ is also quenched, FeCoO${}_{6}$ has a magnetic moment of $3\;\mu_{B}$, despite the high oxidation rate. Thus, Fe–Co alloying produces a magnetic nanoparticle with magnetic moment close (m=3) to or higher (m=1,2,4,5,6) than that of the cobalt oxide dimers, and therefore, mixing is clearly favorable for the smallest Fe–Co nanoalloys.

**Figure 15:**
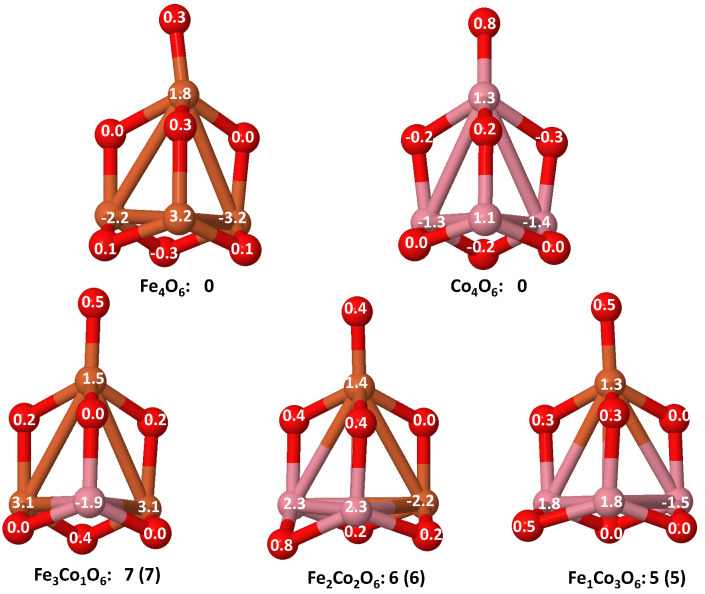
(Color online) Ground states of Fe${}_{4-x}$Co${}_{x}$O${}_{6}$ (*x* = 0–4) oxides. Local magnetic moments of Fe, Co and O atoms are indicated. Positive (negative) numbers indicate spin up (down). Below each of the structures, total magnetic moment is given. Numbers between parentheses are the values of the magnetic excess. Both quantities are in $\mu_{B}$ units. Fe atoms in brown color, Co atoms in pink and oxygen atoms in red.

For larger sizes, n=3 and n=4 (see left panel of Figure 12 and Figure 13), we find a very well defined trend in the regime of low oxidation rates (m<3 for the trimers and m<4 for the tetramers). In this low oxidation rate, the oxidized nanoalloys have a total magnetic moment that lies in between those of the pure Fe and Co oxide clusters (lower than that of Fe${}_{n}$O${}_{m}$ and higher than that of Co${}_{n}$O${}_{m}$). Besides, the larger the relative concentration of Fe with respect to Co, the larger the moment of the oxidized nanoalloy. This is due to two facts: (i) the magnetic couplings are parallel, like in the non oxidized nanoalloy; (ii) the spin polarization in Fe is larger than in Co due to the larger number of d-holes in the former. Thus, from the magnetic point of view, Fe–Co oxidized nanoalloys outperform Co oxide clusters but not Fe oxide clusters, and since no magnetic quenching takes place, the magnetic excess index is close to zero at low oxidation rates. When n=m=3,4, Co oxides have larger magnetic moments than oxidized mixtures. However, at high oxidation rates is where we find that certain oxidized nanoalloys outperform both the pure oxide clusters—in particular, for m=4, m=5 and m=6 in the case of oxidized trimers, and for m=7 in the case of tetramers with the reentrance of the magnetic moment after its quenching in the intermediate oxidation rates. The magnetic excess index exhibits high positive values at those values of *m*, reflecting the outperformance of those nanoalloys. It is interesting to analyze this reentrance of magnetism at m=7 in the oxidized tetramers. Figure 15 shows, for this oxidation rate and for oxidized pure Fe and Co clusters and Fe–Co nanoalloys, the total and local magnetic moments in each one of the Fe, Co and O atoms of the corresponding geometrical structure. Both Fe${}_{4}$O${}_{7}$ and Co${}_{4}$O${}_{7}$ have quenched magnetic moments due to antiparallel couplings. Alloying switches on the reentrance of the magnetic moment, as it changes the magnetic order from antiferromagnetic-like to ferromagnetic-like. Then, once the parallel couplings have been restored, increasing the relative concentration of Fe with respect to Co leads to larger total moments of the oxidized nanoalloy (5 $\mu_{B}$ in Fe${}_{1}$Co${}_{3}$O${}_{7}$, 6 $\mu_{B}$ in Fe${}_{2}$Co${}_{2}$O${}_{7}$, and 7 $\mu_{B}$ in Fe${}_{3}$Co${}_{1}$O${}_{7}$) The ideal, again, is to have as many Fe atoms as possible in the system with P magnetic couplings.

#### 3.2.3. Fe–Ni Oxided Nanoalloys

Ni clusters exhibit magnetic saturation with strong P couplings. Since Ni has even less d-holes than Co in the active valence states, the spin polarization is lower than in Co, which gives rise to smaller local magnetic moments in general. Ni oxide clusters also retain the magnetic moment under oxidation, only being quenched for the largest oxidation rates explored in this work (Ni${}_{3}$O${}_{6}$, Ni${}_{4}$O${}_{7}$) and in Ni${}_{2}$O${}_{3}$, which corresponds to one of the structural anomalies already mentioned in the previous subsection. In fact, this cluster adopts a bent linear structure with exclusively metal–oxygen bonds which imposes indirect Ni–Ni magnetic couplings mediated by O. Nevertheless, the magnetic moments of oxidized Ni dimers exceed or equal those of oxidized Co dimers for m=2,4,5,6, and for larger sizes (n=3 and 4) the same occurs for m=5, and m=5,6, respectively. The magnetic moment of Ni oxide clusters, although low, can be also qualified as robust against oxidation.

Mixing Fe and Ni has a somewhat similar effect as mixing Fe and Co (right panel of Figure 11, Figure 12 and Figure 13). In dimers it is beneficial for most oxidation rates, leading to a rather constant moment of $4\;\mu_{B}$ for m≤3 and m=5, and of $6\;\mu_{B}$ for m=6, values that at least double the moment of the corresponding Ni${}_{2}$O${}_{m}$ and explain the high positive value of the excess magnetic moment ($5\;\mu_{B}$ for m=6). Besides, the magnetic moments of FeNiO${}_{2}$, FeNiO${}_{5}$ and FeNiO${}_{6}$ are $3\;\mu_{B}$ larger than their FeCo counterparts which means that for m=2, m=5 Ni promotes P couplings, but not Co, and for m=6 the promotion of P couplings is larger for Ni. However, for the rest of oxidation rates, m=1,3,4, the magnetic moments of FeCo alloys are higher than those of FeNi ones, although FeNiO${}_{4}$ is in a single state associated with the anomalous structure (already mentioned in the previous subsection) in which no direct Fe–Ni bond exists and thus the indirect Fe–Ni magnetic coupling is mediated by O. In trimers, Fe–Ni alloying only increases the moment at the oxygen concentration m=4, and otherwise the pure Fe oxide clusters perform better. The excess magnetic moment reflects this fact with negative values for most oxidation rates and marked positive values ($4.6\;\mu_{B}$ and $3.3\;\mu_{B}$) at m=4 for both Fe–Ni alloy compositions, but they are higher in the case of Fe${}_{2}$Ni${}_{1}$O${}_{4}$ for the same reason as in Fe–Co nanoalloys: the higher the Fe content with P magnetic couplings in the system, the higher the total moment. In tetramers, alloying is only beneficial for m=4; at an oxidation rate where the Fe–Co mixture did not increase the magnetic character with respect to the corresponding pure oxides; and particularly for m=7 where the explanation is exactly the same as in the oxidized Fe–Co tetramers at m=7 discussed above and related to the quenching of the moment in both Fe${}_{4}$O${}_{7}$ and Ni${}_{4}$O${}_{7}$ and the reentrance of magnetic moment promoted by alloying. We note that m=7 is an oxygen rate at which the structures of the pure and binary TM tetramer oxides are similar, but that this is not essential for the alloying to be beneficial. For instance, alloying is beneficial to both Fe–Ni trimer and tetramer nanoalloys at m=4 despite the fact that nanoalloys have a different structural arrangement from that of the pure TM oxides. The magnetic excess reflects the benefit of alloying particularly for those oxygen rates.

It is worth noting that the Fe–Ni mixture with n=m=2 and ring structure has a magnetic moment of $4\;\mu_{B}$, higher than that of its pure oxides counterparts, leading to a noticeable magnetic excess of $3\;\mu_{B}$. In fact, this particular Fe–Ni nanoalloy is the most favorable among all the single-compound oxides and nanoalloys. For n=m=4, the Fe–Ni mixtures also present higher magnetic moments (of 4 $\mu_{B}$ at x=2, and 6 $\mu_{B}$ at x=3), with high magnetic excess of 3 $\mu_{B}$ and 4.5 $\mu_{B}$, respectively.

#### 3.2.4. Co–Ni Oxided Nanoalloys

Finally, in Co–Ni nanoalloys we mixed two TM elements with marked tendencies towards P magnetic couplings. From the point of view of maximizing the total moment, pure Co oxide clusters are better performing than pure Ni oxide clusters for most oxidation rates except at high oxidation rates (m=5,6 for n=2,4, and m=5 for n=3).

Regarding Co–Ni oxidized nanoalloys, and in the case of dimers, the magnetic moments are higher than those corresponding to both pure oxides for m=2,6 and those of Co oxides for m=5. Positive values of excess magnetic moment result in these oxidation rates, and also for m=3, where the Co–Ni mixture substantially improves the magnetic moment of the corresponding pure antiferromagnetic Ni oxide. Nanoalloys have higher magnetic moments than Ni oxides for most m values (m=1,2,3,6). For trimers and tetramers, the nanoalloys present larger magnetic moments than Ni oxides for all oxidation rates. Moreover, Co–Ni nanoalloys for n=3 again present higher magnetic moments than both pure oxides at high oxidation rates, m=5,6. In addition, the values of excess magnetic moments are high at m=3, particularly for x=2, where the antiferromagnetic character of the Ni${}_{3}$O${}_{3}$ ring is lost due to the presence of Co, due to which a net magnetic moment arises. For tetramers, alloying is beneficial at high oxidation rates, giving rise to high magnetic moments for all nanoalloys (x=2), and to high values of excess of magnetism 4–6 $\mu_{B}$. Therefore, alloying is beneficial for m=2,3,6 in dimers, and at high oxidation rates in the larger nanoalloys (m=5,6 for trimers and m=6,7 for tetramers). The better performance of the nanoalloys for those oxidation rates is reflected in the magnetic excess, particularly at m=6 in dimers (the moment of the oxide nanoalloy is more than twice that of the pure oxide clusters), and m=6,7 in tetramers. At m=7, alloying again switches on the reentrance of the magnetic moment in the oxidized tetramers by promoting parallel couplings, like in the other nanoalloys. We note that in CoNiO${}_{4}$ the magnetic excess is negative (−3). The reason is that it is in a low-spin state with 1 $\mu_{B}$ while both its pure counterparts have 4 $\mu_{B}$. However, there is a nearly degenerated state of CoNiO${}_{4}$ (0.009 eV higher in total energy) in a high-spin state with 5 $\mu_{B}$ and a magnetic excess value of 1 $\mu_{B}$.

It is noteworthy that Co–Ni alloying has an important effect at the equiatomic composition, n=m=2,3,4. These ring structures present an antiferromagnetic character in the cases of pure Fe oxides (0 $\mu_{B}$, 4 $\mu_{B}$, 0 $\mu_{B}$) and pure Ni oxides (2 $\mu_{B}$), and ferromagnetic behavior for pure Co oxides (0 $\mu_{B}$, 9 $\mu_{B}$, 12 $\mu_{B}$). While Fe–Co alloying only increases the magnetic moment by 1 $\mu_{B}$ in the case of n=m=2, and Fe–Ni alloying promotes a ferromagnetic character in the case of n=m=2,4, Co–Ni alloys, whose magnetic moment is equal to 3 $\mu_{B}$ for dimers (n=m=2), higher than their pure counterparts, achieve a ferromagnetic character for trimers (n=m=3) and tetramers (n=m=4), resulting in quite high magnetic moments of 8–7 $\mu_{B}$ and 11–9 $\mu_{B}$, with magnetic excesses of 1.3–2.6 $\mu_{B}$ and 1.5–3–4.5 $\mu_{B}$, respectively.

## 4. Conclusions

We have performed an extensive DFT study of the possibility of inducing ferromagnetic-like order to promote a high-spin state in small TMO-NP (TM = Fe, Co, Ni) by their doping with a different TM elements of the list. We investigated all nanoalloys resulting from the different stoichiometries and with different oxidation rates up to saturation. Our calculations were performed using the SIESTA code [44] with the PBE functional of the GGA to treat the exchange and correlation effects [45].

From the energetic point of view, the binding energies indicate that the higher the concentration of Fe in the TMO-NP, the higher their absolute stability. On the other hand, the higher the Ni content, the lower the exothermic character of oxidation, or in other words, the less prone to oxidation the nanoparticle will be. The critical O concentration beyond which molecular adsorption is energetically favorable as compared to atomic adsorption lowers while going from Fe to Co and Ni. Since molecular adsorption is a manifestation of oxygen saturation of the system, those nanoalloys that are rich in the later TM elements admit less oxygen. Moreover, there is an important correlation between binding energies and electronic charge transfer for all mixtures; low binding energies correlate with large electronic charge transfer to oxygen atoms.

From the structural point of view, oxygen atoms tend to occupy bridge sites between the TM atoms followed by top sites, and preferably bind with Fe atoms, followed by Co and finally Ni. The net electronic charge in the O atoms favors their uniform distribution on the nanoparticle. Weakening of the metal–metal binding while increasing the oxidation rate is manifested in a less compact metal skeleton of the nanoparticle. In a particular case, ring-like structures with O-mediated metal–metal bonds are formed when the number of O atoms equals the number of metal ones, except for Fe${}_{1}$Ni${}_{3}$O${}_{4}$.

Regarding the magnetic properties, a general trend is that an Fe cluster must reach a critical size and metal–metal coordination to retain the parallel magnetic couplings under oxidation. When this happens, since Fe has the highest spin polarization among the considered TMs, we found the TM oxides with the maximum total moments and the maximum stabilities among all oxidized nanoalloys. Unfortunately, another general trend is that the magnetic moment of these small iron nanoparticles is not robust against oxidation and the moment dramatically drops while increasing the oxidation rate due to antiparallel couplings. We have found that combining the stronger tendency of Co and Ni to parallel couplings and the larger spin polarization of Fe (more d-holes) is particularly beneficial for certain nanoalloys in order to achieve a high total magnetic moment and more robust against oxidation. For instance, the oxidized Fe–Co nanoalloys present magnetic moments that exceed at least that of one of their single-compound counterparts, and those of both in the case of dimers. In addition, the larger the relative concentration of Fe with respect to Co, the larger the moment of the oxidized nanoalloy. On the other hand, Co–Ni nanoalloys wherein two TM elements with marked tendencies towards P magnetic couplings are mixed, are robust magnetic grains, with high moments particularly at the equiatomic metal composition. For larger sizes, at high oxidation rates, certain oxidized nanoalloys also outperform both the pure counterparts. In particular, for m=7 in the case of tetramers, alloying of Fe with Co or Ni even promotes the reentrance of magnetism where the pure oxidized counterparts exhibit quenched magnetic moments. This reentrance is due to a change of magnetic order from antiferromagnetic-like to ferromagnetic-like, and once the parallel couplings have been restored, increasing the relative concentration of Fe with respect to Co or Ni leads to higher total moments and higher stability of the oxidized nanoalloy.

We believe that the results reported here may contribute in a better understanding of the mechanisms through which alloying can improve the magnetic figures of merit in an oxidized TM nanoparticle, and thus to help in the design of magnetic TMO-NP for those applications where superparamagnetism is required.

## Figures and Tables

**Table 1:** Binding energies (E${}_{b}$) in eV, bond distance (*d*) in Å and electronic charge transfer (*q*) in *e* units, of the TMO oxides (TM = Fe, Co, Ni) and TM dimers.

	FeO	CoO	NiO	Fe${}_{2}$	Co${}_{2}$	Ni${}_{2}$
E${}_{b}$ (eV)	2.75	2.63	2.29	1.56	1.77	1.35
*d* (Å)	1.67	1.70	1.70	2.04	2.01	2.17
*q* (*e*)	0.46	0.45	0.43	–	–	–

## Supplementary Materials

The following are available online at https://www.mdpi.com/2079-4991/10/9/1814/s1. Figure S1: (Color online) (**a**) Upper panel: average TM–TM distance (green), Co–Co distance (blue) and average Co–Ni distance (red) for Co${}_{2}$Ni${}_{1}$O${}_{m}$ oxides as a function of the number of oxygen atoms, m. (**b**) Lower panel: average TM–O distance (green), average Co-O distance (blue),and average Ni-O distance (red) for Co${}_{2}$Ni${}_{1}$O${}_{m}$ oxides as a function of the number of oxygen atoms, m. Continuous (dashed) lines correspond to average distances between oxygens on bridge (top) positions and TM atoms. Figure S2: (Color online) Binding energies in eV (**upper panel**) and average electronic charge transfer in *e* (**lower panel**) of A${}_{4-x}$B${}_{x}$O${}_{6}$ (A, B = Fe, Co, Ni; x=0−4) oxides. Blue, green and red curves correspond to Fe–Co, Fe–Ni and Co–Ni nanoalloys, respectively.

## References

[1] Lungu I.I., Rǎdulescu M., Mogoşanu G.D., Grumezescu A.M. (2016). pH sensitive core-shell magnetic nanoparticles for targeted drug delivery in cancer therapy. Rom. J. Morphol. Embryol..

[2] Jadhav S., Gaikwad S., Nimse M., Rajbhoj A. (2011). Copper oxide nanoparticles: Synthesis, characterization and their antibacterial activity. J. Clust. Sci..

[3] Qiang Y., Antony J., Sharma A., Nutting J., Sikes D., Meyer D. (2006). Iron/iron oxide core-shell nanoclusters for biomedical applications. J. Nanopart. Res..

[4] Nie S., Xing Y., Kim G.J., Simons J.W. (2007). Nanotechnology applications in cancer. Annu. Rev. Biomed. Eng..

[5] Gupta A.S. (2014). Nanotechnology applications in diagnosis and treatment of metastasis. Nanomedicine.

[6] Azam A., Ahmed A.S., Oves M., Khan M.S., Habib S.S., Memic A. (2012). Antimicrobial activity of metal oxide nanoparticles against Gram-positive and Gram-negative bacteria: A comparative study. Int. J. Nanomed..

[7] Laurent S., Forge D., Port M., Roch A., Robic C., Elst L.V., Muller R.N. (2008). Magnetic iron oxide nanoparticles: Synthesis, stabilization, vectorization, physicochemical characterizations, and biological applications. Chem. Rev..

[8] Jones N., Ray B., Ranjit K.T., Manna A.C. (2008). Antibacterial activity of ZnO nanoparticle suspensions on a broad spectrum of microorganisms. FEMS Microbiol. Lett..

[9] Pankhurst Q.A., Connolly J., Jones S.K., Dobson J. (2003). Applications of magnetic nanoparticles in biomedicine. J. Phys. D Appl. Phys..

[10] Wang H., Cui L.-F., Yang Y., Casalongue H.S., Robinson J.T., Liang Y., Cui Y., Da H. (2010). Mn_3_O_4_ Graphene hybrid as a high-capacity anode material for lithium ion batteries. J. Am. Chem. Soc..

[11] Koo B., Xiong H., Slater M.D., Prakapenka V.B., Balasubramanian M., Podsiadlo P., Johnson C.S., Rajh T., Shevchenko E.V. (2012). Hollow iron oxide nanoparticles for application in lithium ion batteries. Nano Lett..

[12] Wu Z.S., Ren W., Wen L., Gao L., Zhao J., Chen Z., Zhou G., Li F., Cheng H.M. (2010). Graphene anchored with Co_3_O_4_ nanoparticles as anode of lithium ion batteries with enhanced reversible capacity and cyclic performance. ACS Nano.

[13] Stoimenov P.K., Klinger R.L., Marchin G.L., Klabunde K.J. (2002). Metal oxide nanoparticles as bactericidal agents. Langmuir.

[14] Tran M., Mir A., Mallik D., Sinha A., Nayar S., Webster T.J. (2010). Bactericidal effect of iron oxide nanoparticles on Staphylococcus aureus. Int. J. Nanomed..

[15] Sondi I., Salopek-Sondi B. (2004). Silver nanoparticles as antimicrobial agent: A case study on E. coli as a model for Gram-negative bacteria. J. Colloid Interface Sci..

[16] Zhang S., Zhao X., Niu H., Shi Y., Cai Y., Jiang G. (2009). Superparamagnetic Fe_3_O_4_ nanoparticles as catalysts for the catalytic oxidation of phenolic and aniline compounds. J. Hazard. Mater..

[17] Jiao F., Frei H. (2009). Nanostructured cobalt oxide clusters in mesoporous silica as efficient oxygen evolving catalysts. Angew. Chem..

[18] Teja A.S., Koh P.-Y. (2009). Synthesis, properties, and applications of magnetic iron oxide nanoparticles. Prog. Cryst. Growth Charact. Mater..

[19] Carnes C.L., Klabunde K.J. (2003). The catalytic methanol synthesis over nanoparticle metal oxide catalysts. J. Mol. Catal. A Chem..

[20] Lopez N., Norskov J.K. (2002). Catalytic CO oxidation by a gold nanoparticle: A density functional study. J. Am. Chem. Soc..

[21] Johnson G.E., Reilly N.M., Castleman A.W. (2009). Effect of charge state and stoichiometry on the structure and reactivity of nickel oxide clusters with CO. Int. J. Mass Spectrom..

[22] Wan J., Yuan R., Zhang C., Wu N., Yan F., Yu S., Chen K. (2016). Potential application of metal dichalcogenides double-layered heterostructures as anode materials for Li-ion batteries. J. Phys. Chem. C.

[23] Casula M.F., Conca E., Bakaimi I., Sathya A., Materia M.E., Casu A., Falqui A., Sogne E., Pellegrino T., Kanaras A.G. (2016). Manganese doped-iron oxide nanoparticle clusters and their potential as agents for magnetic resonance imaging and hyperthermia. Phys. Chem. Chem. Phys..

[24] Szczerba W., Zukrowski J., Przybylski M., Sikora M., Safonova O., Shmeliov A., Nicolosi V., Schneider M., Granath T., Oppmann M. (2016). Pushing up the magnetisation values for iron oxide nanoparticles via zinc doping: X-ray studies on the particle’s sub-nano structure of different synthesis routes. Phys. Chem. Chem. Phys..

[25] Wang Y., Chen Q., Wang J. (2012). Ab initio study of structure and magnetism of late transition metal oxide TM*_n_*O*_m_* (TM = Fe, Co, Ni, n = 1, 2, m = 1–6). J. Nanosci. Nanotechnol..

[26] Erlebach A., Hunhn C., Jana R., Sierka M. (2014). Structure and magnetic properties of (Fe_2_O_3_)*_n_* clusters (*n* = 1–5). Phys. Chem. Chem. Phys..

[27] Reilly N.M., Reveles J.U., Johnson G.E., Khanna S.N., Castelman A.W. (2007). Experimental and theoretical study of the structure and reactivity of Fe_1−2_$O_{\leq 6}^{-}$ clusters with CO. J. Phys. Chem. A.

[28] Reilly N.M., Reveles J.U., Johnson G.E., del Campo J.M., Khanna S.N., Köster A.M., Castleman A.W. (2007). Experimental and theoretical study of the structure and reactivity of Fe*_m_*O*_n_*^+^ (*m* = 1,2; *n* = 1–5) with CO. J. Phys. Chem. C.

[29] Ota K., Koyasu K., Ohshimo K., Misaizu F. (2013). Structures of cobalt oxide cluster cations studied by ion mobility mass spectrometry. Chem. Phys. Lett..

[30] Tung N.T., Tam N.M., Nguyen M.T., Lievens P., Janssens E. (2014). Influence of Cr doping on the stability and structure of small cobalt oxide clusters. J. Chem. Phys..

[31] Johnson G.E., Reveles J.U., Reilly N.M., Tyo E.C., Khanna S.N., Castelman A.W. (2008). Influence of stoichiometry and charge state on the structure and reactivity of cobalt oxide clusters with CO. J. Phys. Chem. A.

[32] Kumavat S., Deshpande M. (2014). Alkali metal doped nickel oxide clusters: A density functional study. Comput. Theor. Chem..

[33] Yin S., Xue W., Ding X.-L., Wang W.-G., He S.-G., Ge M.-F. (2009). Formation, distribution, and structures of oxygen-rich iron and cobalt oxide clusters. Int. J. Mass Spectrom..

[34] Dible C.J., Akin S.T., Ard S., Fowler C.P., Duncan M.A. (2012). Photodissociation of cobalt and nickel oxide cluster cations. J. Phys. Chem. A.

[35] Kirilyuk A., Fielicke A., Demyk K., von Helden G., Meijer G., Rasing T.H. (2010). Ferrimagnetic cagelike Fe_4_O_6_ cluster: Structure determination from infrared dissociation spectroscopy. Phys. Rev. B.

[36] Li S., Zhai H.-J., Wang L.-S., Dixon D.A. (2012). Structural and electronic properties of reduced transition metal oxide clusters, M_4_O_10_ and M_4_O_10_^−^ (M = Cr, W), from photoelectron spectroscopy and quantum chemical calculations. J. Phys. Chem. A.

[37] Wang H.-Q., Li H.-F. (2012). Probing the structural and electronic properties of small vanadium dioxide clusters by density functional theory and comparison with experimental photoelectron spectroscopy. J. Chem. Phys..

[38] Ohshimo K., Komukai T., Moriyama R., Misaizu F. (2014). Isomer separation of iron oxide cluster cations by ion mobility mass spectrometry. J. Phys. Chem. A.

[39] Aguilera-del-Toro R.H., Aguilera-Granja F., Vega A., Balbás L.C. (2014). Structure, fragmentation patterns, and magnetic properties of small cobalt oxide clusters. Phys. Chem. Chem. Phys..

[40] Aguilera-del-Toro R.H., Aguilera-Granja F., Balbás L.C. (2017). Structure, fragmentation patterns, and magnetic properties of small nickel oxide clusters. Phys. Chem. Chem. Phys..

[41] Aguilera-del-Toro R.H., Aguilera-Granja F., Torres M.B., Vega A. Relation between structural patterns and magnetism in small iron oxide clusters; reentrance of magnetic moment at hich oxidation rates.

[42] Torres M.B., Aguado A., Aguilera-Granja F., Vega A., Balbás L.C. (2015). Structural, vibrational, and magnetic properties of FeCoO_*n*_^0/+^ (*n* = 1–6) bimetallic oxide clusters. Phys. Chem. C.

[43] Borzorth R.M. (1993). Ferromagnetism.

[44] Soler J.M., Artacho E., Gale J.D., García A., Junquera J., Ordejón P., Sánchez-Portal D. (2002). The SIESTA method for ab initio order-N materials simulation. J. Phys. Condens. Matter.

[45] Perdew J.P., Burke K., Ernzerhof M. (1996). Generalized gradient approximation made simple. Phys. Rev. Lett..

[46] Troullier N., Martins J.L. (1991). Efficient pseudopotentials for plane-wave calculations. Phys. Rev. B.

[47] Kleinman L., Bylander D.M. (1982). Efficacious form for model pseudopotentials. Phys. Rev. Lett..

[48] Louie S.G., Froyen S., Cohen M.L. (1982). Nonlinear ionic pseudopotentials in spin-density-functional calculations. Phys. Rev. B.

[49] Pauling L. (1960). The Nature of the Chemical Bond.

[50] Aguilera-del-Toro R.H., Aguilera-Granja F., Balbás L.C., Vega A. (2018). Synthesis and characterization of indium oxide nanoparticles. Theor. Chem. Acc..

[51] Ohshimo K., Azuma A., Komukai T., Moriyama R., Misaizu F. (2015). Structures and CO-adsorption reactivities of nickel oxide cluster cations studied by ion mobility mass spectrometry. J. Phys. Chem. C.

[52] Fritsch D., Koepernik K., Richter M., Eschrig H. (2007). Transition metal dimers as potential molecular magnets: A challenge to computational chemistry. J. Comput. Chem..

[53] Bloński P., Hafner J. (2009). Magnetic anisotropy of transition-metal dimers: Density functional calculations. Phys. Rev. B.

[54] Bloński P., Hafner J. (2011). Magneto-structural properties and magnetic anisotropy of small transition-metal clusters: A first-principles study. J. Phys. Condens. Matter.

[55] Fortunelli A., Velasco A.M. (1999). Structural and electronic properties of Pt/Fe nanoclusters from EHT calculations. J. Mol. Struct. Theochem.

[56] Aguado A., López J.M. (2011). Identifying structural and energetic trends in isovalent core-shell nanoalloys as a function of composition and size mismatch. J. Chem. Phys..

