# Supplementary material for: Tuning the Magnetic Moment of Small Late 3d-Transition-Metal Oxide Clusters by Selectively Mixing the Transition-Metal Constituents
Source: Nanomaterials (Basel). 2020 Sep 11;10(9):1814. doi: 10.3390/nano10091814 (PMC7559123; doi:10.3390/nano10091814)
Supplement: Supplementary file 1 [file nanomaterials-10-01814-s001.pdf]

Article

Electronic Suplementary Information for the article *"Tuning the Magnetic Moment of Small Late 3d-Transition-Metal Oxide Clusters by Selective Mixing the Transition-Metal Constituents."*

Rodrigo H. Aguilera-del-Toro[1], María B. Torres[2,*], Fausino Aguilera-Granja[3] and Andrés Vega[1]

[1] Departamento de Física Teórica, Atómica y Óptica, Universidad de Valladolid, Valladolid 47011, Spain; rodrigohumberto.aguilera@uva.es (R.H.A.-d.-T.); avega@fta.uva.es (A.V.)
[2] Departamento de Matemáticas y Computación, Universidad de Burgos, Burgos 09006, Spain; begonia@ubu.es (M.B.T.)
[3] Instituto de Física, Universidad Autónoma de San Luis Potosí, San Luis Potosí 78290, México; faustino@ifisica.uaslp.mx (F.A.-G.)
* Correspondence: begonia@ubu.es

Version September 10, 2020 submitted to Nanomaterials

Keywords: magnetism; transition-metal oxide clusters; DFT calculations; structure; electronic properties;

PACS: 75.75+a; 36.40Cg; 75.30.Pd; 75.50.-y

© 2020 by the authors. Submitted to *Nanomaterials* for possible open access publication under the terms and conditions of the Creative Commons Attribution (CC BY) license (http://creativecommons.org/licenses/by/4.0/).

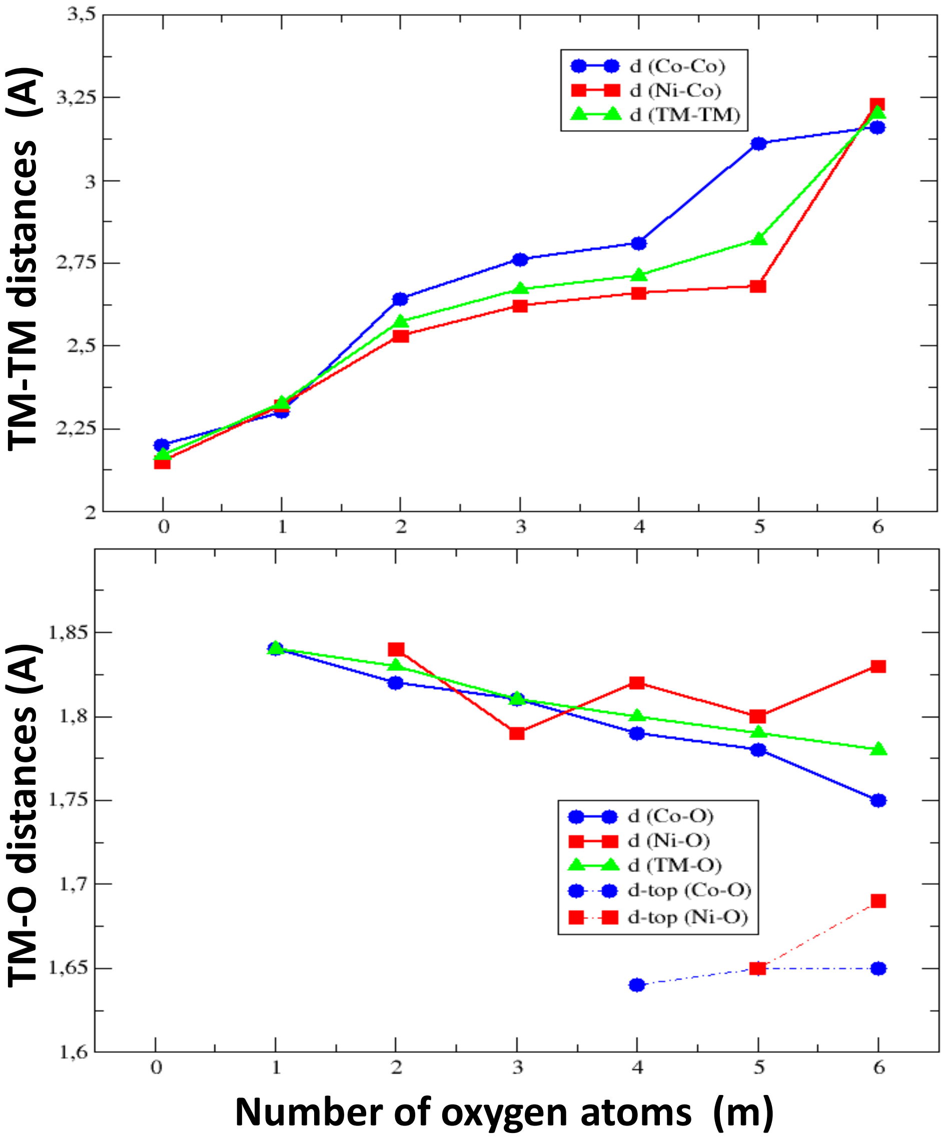

Figure 1. (Color online) (**a**) Upper panel: average TM-TM distance (green), Co-Co distance (blue), and average Co-Ni distance (red) for $Co_2Ni_1O_m$ oxides as a function of the number of oxygen atoms, m. (**b**) Lower panel: average TM-O distance (green), average Co-O distance (blue), and average Ni-O distance (red) for $Co_2Ni_1O_m$ oxides as a function of the number of oxygen atoms, m. Continuous (dashed) lines correspond to average distances between oxygens on bridge (top) positions and TM atoms.

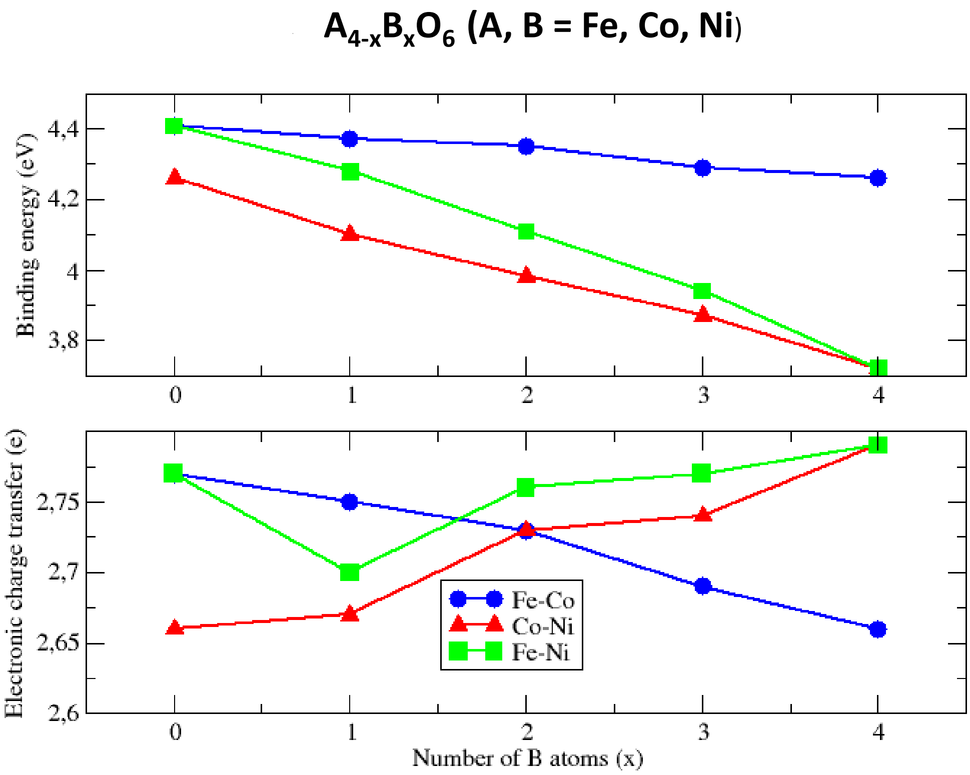

Figure 2. (Color online) Binding energies in eV (upper panel) and average electronic charge transfer in e (lower panel) of $A_{4-x}B_xO_6$ (A, B = Fe, Co, Ni; $x = 0 - 4$) oxides. Blue, green and red curves correspond to Fe-Co, Fe-Ni and Co-Ni nanoalloys, respectively.
